# When A+B < A: Cognitive Bias in Experts’ Judgment of Environmental Impact

**DOI:** 10.3389/fpsyg.2018.00823

**Published:** 2018-05-29

**Authors:** Mattias Holmgren, Alan Kabanshi, John E. Marsh, Patrik Sörqvist

**Affiliations:** ^1^Department of Building, Energy and Environmental Engineering, University of Gävle, Gävle, Sweden; ^2^School of Psychology, University of Central Lancashire, Preston, United Kingdom

**Keywords:** averaging bias, judgment, environmental impact, climate change, negative footprint illusion

## Abstract

When ‘environmentally friendly’ items are added to a set of conventional items, people report that the total set will have a lower environmental impact even though the actual impact increases. One hypothesis is that this “negative footprint illusion” arises because people, who are susceptible to the illusion, lack necessary knowledge of the item’s actual environmental impact, perhaps coupled with a lack of mathematical skills. The study reported here addressed this hypothesis by recruiting participants (‘experts’) from a master’s program in energy systems, who thus have bachelor degrees in energy-related fields including academic training in mathematics. They were asked to estimate the number of trees needed to compensate for the environmental burden of two sets of buildings: one set of 150 buildings with conventional energy ratings and one set including the same 150 buildings but also 50 ‘green’ (energy-efficient) buildings. The experts reported that less trees were needed to compensate for the set with 150 conventional and 50 ‘green’ buildings compared to the set with only the 150 conventional buildings. This negative footprint illusion was as large in magnitude for the experts as it was for a group of novices without academic training in energy-related fields. We conclude that people are not immune to the negative footprint illusion even when they have the knowledge necessary to make accurate judgments.

## Introduction

Climate change is one of society’s great challenges (American Psychological Association, 2008; Hansen et al., 2013). The scientific community agrees that human activity is to a large degree responsible for these changes (Oreskes, 2005), reinforced by a range of psychological phenomena including justification of environmentally irresponsible behavior with other credentials (Mazar and Zhong, 2010), compensatory green beliefs (Kaklamanou et al., 2015) and rebound effects (Chitnis et al., 2013). People travel for longer distances when driving a vehicle that uses a ‘sustainable’ energy source; they purchase ‘organic’ food as a means to be environmentally friendly without necessarily reducing other means of consumption; and those who deliberately change their behavior to more environmentally friendly in one area often start behaving environmentally irresponsible in another. Psychological science may not be able to stop climate change on its own (Gifford, 2011) but it can help identify why and how people’s ability to make accurate environmental impact estimates of their actions is biased (Sörqvist, 2016). Drawing from previous research (Holmgren et al., 2018), this paper investigates whether the estimated environmental impact of a set of items is reduced when “green” objects are added to the set—a negative footprint illusion. Specifically, the current study explores whether people are immune to this illusion when they have the necessary knowledge to make accurate estimates.

A large part of psychological research on erroneous ways of reasoning has concerned probability judgment. This research tradition has identified a large body of heuristics and biases that influence judgments and often lead to inaccuracies (Tversky and Kahneman, 1983; Gilovich et al., 2002; Isaac and Brough, 2014; Gigerenzer and Gaissmaier, 2015). Here, we apply these concepts and methods on the study of people’s understanding of environmental impact. Past studies with similar ambitions have shown, for example, that people tend to rely on symbolically significant information (Sütterlin and Siegrist, 2014) or other irrelevant attributes such as object size (Cowen and Gatersleben, 2017) while neglecting other more important information when judging energy use. People also have difficulty understanding the stock-flow relationship of the CO_2_ accumulation in the atmosphere (Newell et al., 2016); and they believe that environmentally friendly actions and choices can compensate for less sustainable behaviors (Kaklamanou et al., 2015). The latter of these conceptions is called ‘compensatory green beliefs’ and may be one of the reasons for negative spillover of pro-environmental behavior. Negative spillover occurs when “one pro-environmental behavior decreases the likelihood of additional pro-environmental behavior” (Truelove et al., 2014, p. 128). For example, fuel efficiency is associated with increased driving distance (Matiaske et al., 2012), the presence of a recycling bin increases paper use compared to a control condition (Catlin and Wang, 2012), and car owners of more environmentally friendly cars drive more unethically compared to conventional car owners (Norton, 2012). These negative spillover effects can be linked to moral licensing whereby people justify immoral behavior by establishing moral credentials (Mazar and Zhong, 2010). One possible explanation for this phenomenon – as proposed by Sachdeva et al. (2009) – is that moral behavior is inherently costly for the individual; hence they use moral licensing to get back to a more comfortable state—that is, a regression to the individual average of the ‘moral currency.’

Compensatory green beliefs are often associated with behavior; the idea that the benefits of some behaviors can compensate the costs of other behaviors. A similar phenomenon has also been found in people’s tendency to think about the costs and benefits of objects. Holmgren et al. (2018) showed that people’s estimates of the environmental impact of a set of conventional buildings are higher, compared to a set containing the same number of conventional buildings in addition to a number of “green” (energy efficient) buildings, as if the benefits of the “green” buildings compensate for some of the costs of the conventional buildings. Experimental evidence suggests that an averaging process underpins this “negative footprint illusion,” by which people base their estimates on the average rather than the sum of the items in the set. We argue that this averaging process is responsible for people’s tendency to think that the addition of environmentally friendly objects compensates for the negative impact of less friendly objects (Holmgren et al., 2018). Further, we assume that the averaging bias has its roots in a balancing heuristic shaped by natural selection to handled social exchange between people (Sörqvist and Langeborg, unpublished). The balancing heuristic is arguably designed to calculate the moral balance in interpersonal relationships and works well when applied to the problem it was designed to solve, but the same principle leads to inaccuracies when applied to estimate the environmental impact of objects and actions.

A negative footprint illusion has also been found in other contexts. Gorissen and Weijters (2016) demonstrated the effect in three experiments on estimates of the environmental impact of food products. In one of their experiments, people rated a hamburger together with an organic apple as having a lower carbon footprint (i.e., environmental impact) compared to the hamburger alone. Collectively, the studies on the negative footprint illusion suggest that the effect influences estimates generally and is not paradigm specific. It should be noted though, that the effect has thus far only been found in between-participants designs in which one group of participants make the environmental impact estimates of the set with regular items and another group of participants make the estimates of the set with regular and “green” items. The illusion has not been shown in within-participant designs where the same participants make estimates of both sets and thus can compare their own estimates of the two. Finding a negative footprint illusion in a within-participant design is both theoretically and methodologically relevant, however, because it would suggest that people are not immune to the illusion when they compare their estimates and, furthermore, such a design can be used to efficiently and reliably analyze individual differences in susceptibility to the illusion.

Another question relating to generalizability is whether expertise modulates the negative footprint illusion. One hypothesis is that the negative footprint illusion arises because people who are susceptible to the illusion lack necessary knowledge of the item’s actual environmental impact, perhaps in combination with a lack of mathematical skills. Thus, people who are used to approaching problems with mathematical tools, and have an understanding of the item’s actual environmental impact that goes beyond the layperson’s, might be less likely to fall prey to the influence of the balancing heuristic, and instead more accurately arrive at the sum of the environmental impact of the items rather than their average. By definition, experts often outperform novices (e.g., Ericsson and Lehmann, 1996). This is typically also the case in the context of judgment and decision-making (Spence and Brucks, 1997; Kuusela et al., 1998) and consequently people often trust experts in making high quality decisions and many let experts make choices for them, for example allowing them to handle their stock-portfolios and funds. However, there are cases where training and practice does not lead to better performance. Research has demonstrated that recognition based portfolios (i.e., the set of most-recognized options) outperform portfolios managed by stock-experts (Ortmann et al., 2008). Correspondingly, there is a large body of research suggesting that experts are little, if at all, better than novices at making accurate judgments. For example, experts as well as novices are susceptible to violations of the conjunction rule (i.e., A < A+B; Tversky and Kahneman, 1983), and experts are good at identifying components necessary for accurate judgments but are poor at combining those components (Newell et al., 2015).

The purpose of the present paper was to investigate whether expertise modulates the magnitude of the negative footprint illusion. To this end, energy systems graduate students—with academic degrees in energy-related fields and academic training in mathematics—were recruited as participants and asked to estimate the environmental impact of buildings with varying energy ratings. Thus, “experts” in the current context refers to people with the necessary knowledge and training in energy systems engineering and its relationship to environmental impact. To further explore the generalizability of the negative footprint illusion, the study was also designed to challenge the hypothesis that the effect disappears when people are able to compare the two sets of to-be-estimated objects (i.e., in a within-participants design). We implemented a new judgmental dimension. Instead of requesting participants to estimate the ‘carbon footprint’ of the two sets, one set with only conventional items (A) and another set with an addition of “green” items (A+B), the participants of the current study were asked how many trees would be necessary to compensate for the two sets’ greenhouse gas emissions due to energy use. We hypothesized that the participants would estimate that more trees are necessary to compensate for a set of conventional buildings (A) in comparison with a set of conventional and “green” buildings (A+B), as they would erroneously think that A + B < A.

## Materials and Methods

### Participants

A total of 55 participants (42% women), 22 energy system graduates (henceforth called experts) and 33 undergraduate students at the University of Gävle without formal education in environmental issues or energy engineering (henceforth called novices) participated in the experiment (mean age = 27.58, *SD* = 6.18). The study was conducted in accordance with the declaration of Helsinki and the ethical guidelines given by the American Psychological Association. All participants were adults and participated with informed consent. The participants signed an information agreement form. The study did not treat sensitive personal data, did not entail a physical intervention or methods with the purpose of affecting a research person, the data collection did not include apparent risk of injury, and no data could or can be traced to individual persons. Because of this, no external ethical review was requested for the study reported here in accordance with Swedish law.

### Materials

A questionnaire was used to obtain data. On the first page of the questionnaire, participants were told that they were about to make two evaluations and were asked to carefully read the information before making the evaluations. In one condition, the participants viewed an abstract representation of a suburb consisting of 150 ‘conventional’ buildings with appurtenant energy efficiency ratings, and they were told that all units had the same materials and performance characteristics. In the other condition, the participants viewed an identical suburb, with the exception that another 50 ‘green’ (i.e., environmentally certified) buildings with appurtenant energy efficiency ratings were also present (i.e., a total of 200 buildings). For each condition, the participants were given the following information before making the evaluation: *“Energy use is linked to greenhouse gas (GHG) emissions, which in turn are harmful for the environment. For example, assume for every unit of electrical energy used and for every unit of residential heat used, a certain amount of CO_2_ is produced. A transportation company based in Örebro, has introduced an environmental compensation policy, that is, if one of their cars covers 33,000 km, the company will plant 50 trees. Planted trees absorb CO_2_ for many years, and can therefore compensate for the negative environmental consequences of increased energy use.”* They were then asked to estimate how many trees on a scale from 1 to 100 each suburb needed to compensate for their monthly energy use. The two conditions were counterbalanced between participants.

### Design and Procedure

A within-between mixed participants design was used with two independent variables. One independent variable was ‘suburb type,’ manipulated within participants in two conditions: 150 conventional buildings (henceforth called the ‘conventional condition’) vs. 150 conventional buildings with an addition of 50 “green” buildings (henceforth called the ‘green addition condition’). The order between the two suburb types was counterbalanced between participants. More specifically, half of the participants were randomly assigned to begin with the green addition condition and the other half were assigned to begin with the conventional condition. The other independent variable was ‘group type’: 33 participants belonged to the novice group, whereas 22 participants belonged to the experts group.

## Results

**FIGURE 1:**
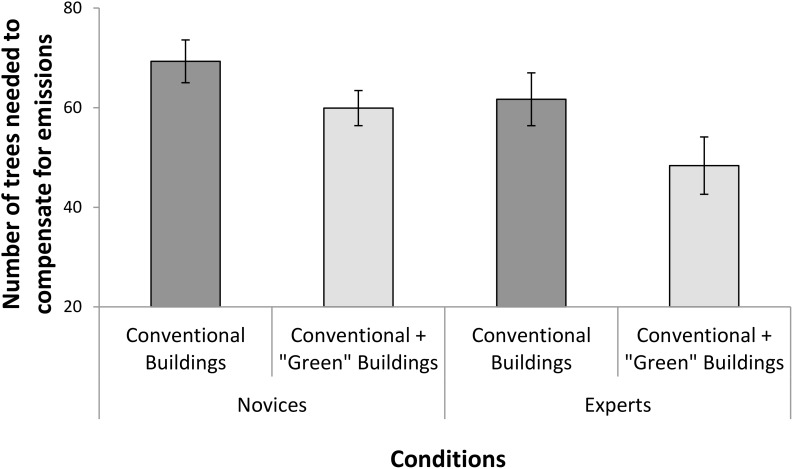
Experts’ (energy system graduates) and novices’ estimates of how many trees are needed to compensate for the energy use of a community with 150 ‘conventional’ buildings and a community with 150 ‘conventional’ and 50 ‘green’ buildings. Estimates appear to be underpinned by an averaging bias by which people believe that the addition of more ‘environmentally friendly’ objects compensates for the negative impact of less friendly objects. Error bars represent standard error of means.

As can be seen in **Figure 1**, both the experts and the novices reported that fewer trees were needed for the suburb with ‘conventional’ and ‘green’ buildings compared to the condition with only the ‘conventional’ buildings. This was confirmed by a 2 (suburb type: conventional condition vs. green addition condition) × 2 (group type: novices vs. experts) mixed analysis of variance, which revealed a main effect of suburb type, *F*(1,53) = 10.27, *p* = 0.002, $\eta_{p}^{2}$ = 0.16. The analysis did not reveal a significant interaction, *F*(1,53) = 0.31, *p* = 0.582, $\eta_{p}^{2}$ = 0.01, nor a main effect of group, *F*(1,53) = 2.97, *p* = 0.091, $\eta_{p}^{2}$ = 0.05, showing that experts and novices responded similarly.

Because this conclusion rests on a null-hypothesis, we also report the Bayesian factors (BFs) for the effects. The BF01 for the main effect of suburb type was 0.057, *p*(H0—D) = 0.053, *p*(H1—D) = 0.946, which is regarded as positive evidence of the alternative hypothesis (Masson, 2011). The BF01 for the interaction was 6.32, *p*(H0—D) = 0.863, *p*(H1—D) = 0.137, positive evidence for the null hypothesis, and the BF01 for the main effect of group was 1.01, *p*(H0—D) = 0.623, *p*(H1—D) = 0.377, weak evidence for the null hypothesis. The Bayesian analyses reinforce the conclusion that experts were susceptible to the negative footprint illusion and to a degree similar to that of novices.

## Discussion

The present study shows that when energy-efficient buildings are added to a set of less energy-efficient buildings, the estimated environmental impact of the total set is *reduced* rather than *increased*. The results revealed a highly reliable manifestation of this illusion with no difference between novices and experts. Because of this, we conclude that people can be susceptible to the negative footprint illusion even when they have sufficient mathematical skills and knowledge of the items’ environmental impact. Moreover, the present study also suggests that the illusion can survive when people are able to compare the two to-be-evaluated sets and generalizes to judgment dimensions other than carbon footprint estimates (Gorissen and Weijters, 2016; Holmgren et al., 2018).

While level of expertise made no difference to the negative footprint illusion reported here, it should, however, be mentioned that there are research domains within which experts indeed outperform novices at making accurate decisions (Spence and Brucks, 1997; Kuusela et al., 1998). A group of energy system graduates were chosen as experts in this particular experiment, because the estimates they were asked to make concerned compensation for buildings’ energy use. In contrast to novices, energy system graduates had expert knowledge of this issue. Furthermore, this group of ‘graduate students’ should be viewed as experts in contrast to the group of novices with regards to buildings energy systems and environmental assessments because these subjects are extensively covered in the course work of their graduate program. The ‘experts’ in the current study are not necessarily representative of energy-system researchers or others with extensive experience of the energy profession, who perhaps more justly could be called “true” experts in the field. However, we conclude that the negative footprint illusion is seemingly resilient to some levels of expertise, including university training in mathematics and at least to a basic understanding of the to-be-estimated objects’ actual environmental impact.

A possible explanation of the negative footprint illusion, based on previous research (Holmgren et al., 2018), is that the effect is underpinned by an averaging process. Rather than basing their estimates of the environmental impact of a category of items on the sum of A and B (which would be more accurate), people appear to base their estimates on the average of A and B. The estimator calculates (probably unconsciously) the average of the items in the set, whereby the “green” objects in the set contribute to the average with lower values, whereas the conventional objects contribute with higher values. The environmental impact estimate that results from this process follows a principle that can be expressed as A + B < A, whereby A are conventional items and B are environmentally friendly items. This erroneous way of reasoning has been termed “the averaging bias” (Holmgren et al., 2018) and could be responsible for people’s false belief that “green” objects actually benefit the environment when in fact they are simply ‘less bad’ than their conventional counterparts. On this view, an alternative explanation of the negative footprint illusion is that participants more simply assess the sum of conventional items and “green” items as A – B, where conventional items are viewed as ‘bad’ for the environment and “green” items as ‘good’ (or beneficial) to the environment. However, past research shows that people assess “green” items as environmentally harmful, but to a lesser degree than conventional items, why the averaging explanation of the negative footprint illusion appears to be more likely.

Our supposition is that the averaging bias may pervade many contexts and behavioral settings wherein people explicitly and implicitly evaluate a mixture of objects and actions, such as choosing consumer goods and estimating the health of food products (Chernev and Gal, 2010) and the benefits of emission cuts (Lewandowsky, 2016). The averaging bias may also underpin the belief that undertaking environmentally friendly actions can compensate for less friendly ones (Kaklamanou et al., 2015).

### Applied Implications

This study – which suggests that the illusion manifests also when people are able to compare the two to-be-evaluated sets – strengthens the case that this effect can have negative consequences for the environment when people make environment-related decisions. For example, consumers may choose to purchase eco-labeled food products as a way to compensate for buying conventional products, with the intention to decrease the environmental impact of their consumer behavior, when they are – in fact – increasing the burden by adding more products regardless of the eco-label. This stresses the need to find methods that can limit the tendency to base impact estimates on the average of conventional products and “green” products (?).

Another real world situation in which the negative footprint illusion can have negative consequences for the environment concerns management decision-making, for example in the context of urban planning. If experts within disciplines that have a direct influence on environmental management – implicitly or explicitly – think “green” buildings have the ability to decrease the carbon footprint of a community (as this study suggests), then it is essential to reconsider how “green” buildings are portrayed in various discourses. The way that “green” buildings appear to be represented in people’s minds at present could lead decision makers and policy makers to promote the construction of “green” buildings for the wrong reasons, believing that the “green” buildings will reduce the environmental burden of a community rather than adding further to it.

## Author Contributions

MH, AK, and PS generated the idea and study design. MH and AK collected and analyzed the data. MH, AK, JM, and PS wrote the paper.

## Conflict of Interest Statement

The authors declare that the research was conducted in the absence of any commercial or financial relationships that could be construed as a potential conflict of interest.
